# Racial discrimination and health: a prospective study of ethnic minorities in the United Kingdom

**DOI:** 10.1186/s12889-020-09792-1

**Published:** 2020-11-18

**Authors:** Ruth A. Hackett, Amy Ronaldson, Kamaldeep Bhui, Andrew Steptoe, Sarah E. Jackson

**Affiliations:** 1 Health Psychology Section, Institute of Psychiatry, Psychology and Neuroscience, King’s College London, London, UK; 2 Department of Behavioural Science and Health, University College London, London, UK; 3 Department of Psychological Medicine, Institute of Psychiatry, Psychology and Neuroscience, King’s College London, London, UK; 4 Centre for Department of Psychiatry & Nuffield Department of Primary Care Health Sciences, University of Oxford, Oxford, UK

**Keywords:** Racism, Discrimination, Prejudice, Mental health, Physical health

## Abstract

**Background:**

Racism has been linked with poor health in studies in the United States. Little is known about prospective associations between racial discrimination and health outcomes in the United Kingdom (UK).

**Methods:**

Data were from 4883 ethnic minority (i.e. non-white) participants in the UK Household Longitudinal Study. Perceived discrimination in the last 12 months on the basis of ethnicity or nationality was reported in 2009/10. Psychological distress, mental functioning, life satisfaction, self-rated health, physical functioning and reports of limiting longstanding illness were assessed in 2009/10 and 2011/12. Linear and logistic regression analyses adjusted for age, sex, income, education and ethnicity. Prospective analyses also adjusted for baseline status on the outcome being evaluated.

**Results:**

Racial discrimination was reported by 998 (20.4%) of the sample. Cross-sectionally, those who reported racial discrimination had a greater likelihood on average of limiting longstanding illness (odds ratio (OR) = 1.78, 95% confidence interval (CI) 1.49; 2.13) and fair/poor self-rated health (OR = 1.50; 95% CI 1.24; 1.82) than those who did not report racial discrimination. Racial discrimination was associated with greater psychological distress (*B* = 1.11, 95% CI 0.88; 1.34), poorer mental functioning (*B* = − 3.61; 95% CI -4.29; − 2.93), poorer physical functioning (*B* = − 0.86; 95% CI -1.50; − 0.27), and lower life satisfaction (*B* = − 0.40, 95% CI -0.52; − 0.27). Prospectively, those who reported racial discrimination had a greater likelihood on average of limiting longstanding illness (OR = 1.31, 95% CI 1.01; 1.69) and fair/poor self-rated health (OR = 1.30; 95% CI 1.00; 1.69), than those who did not report racial discrimination. Racial discrimination was associated increased psychological distress (*B* = 0.52, 95% CI 0.20; 0.85) and poorer mental functioning (*B* = − 1.77; 95% CI -2.70; − 0.83) over two-year follow-up, adjusting for baseline scores.

**Conclusions:**

UK adults belonging to ethnic minority groups who perceive racial discrimination experience poorer mental and physical health than those who do not. These results highlight the need for effective interventions to combat racial discrimination in order to reduce inequalities in health.

**Supplementary Information:**

The online version contains supplementary material available at 10.1186/s12889-020-09792-1.

## Background

Discrimination is defined as the differential treatment of an individual based on a socially ascribed characteristic [1]. In the United Kingdom (UK), the 1965 Race Relations Act [2] outlawed discrimination on the grounds of colour, nationality and ethnic or national origins. Race remains a protected characteristic under contemporary equality law [3]. Despite this legislative effort, ethnic inequalities in education, work, health and criminal justice remain [4].

Discrimination on the basis of ethnic origin is regarded as the most common type of prejudice in Europe, with 64% of adults perceiving racial discrimination to be widespread in a survey of 27,718 people [5]. In Britain in 2017, 26% of a representative sample described themselves as racially prejudiced [6], and race continues to be the most common motivator for hate crime incidents [7, 8]. Against the backdrop of the vote to leave the European Union (Brexit), hostility towards migrants and the growth in right-wing nationalist movements [9], these figures reflect a rise in reported racial discrimination in both the UK and Europe [5, 6].

A growing body of research has investigated discrimination as a determinant of mental health [10–12] and to a lesser extent physical health [11]. In an early meta-analysis of 110 studies, discrimination was linked with poor mental health, including psychological distress and decreased life satisfaction [11]. A sub-set of 36 studies in the review investigated associations with physical health. Significant associations were detected in a pooled analysis with various outcomes including hypertension and acute cardiovascular responses to laboratory discrimination protocols. A more recent meta-analysis of 328 studies focusing on discrimination and mental health outcomes alone, again observed that those who perceived discrimination had poorer mental health [12]. This finding was also detected in an independent analysis of 211 cross-sectional studies linking racial discrimination with poor mental health [12].

Racism is a recognised social determinant of health and a driver of ethnic inequities in health [13]. It can be understood as a complex, organised system embedded in socio-political and historical contexts, that involves classifying ethnic groups into social hierarchies. These groups are ideologically assigned differential value, which drives disparities in access to power, resources and opportunities [14, 15]. It occurs at both structural and individual levels (self-reported experiences of racial discrimination) [14, 15].

Several reviews and meta-analyses have focused solely on perceived racial discrimination and health outcomes [13, 16–18]. The largest study to date meta-analysed the results from 293 studies and assessed both mental and physical health outcomes [16]. In this analysis, racial discrimination was associated with poorer overall mental health including greater psychological distress, poorer life satisfaction and poorer general mental functioning in independent analyses. Racism was also linked with poorer general health and poorer physical health overall, though few effects remained significant when looking at specific physical health outcomes in separate analyses.

Racial discrimination at the structural and individual level is theorised to impact health through several mechanisms [15]. At the structural level racial discrimination may operate through the unfair allocation of societal resources that are determinants of health (e.g. education, employment, housing) [14, 15] and through differential access to healthcare, as well as perceived poorer quality of care [19]. Another mechanism linking racial discrimination and health could be through the dysregulation of stress-related biological processes [20]. Frequent exposure to racial discrimination is a chronic stressor and has been linked with dysregulated cardiovascular, neuroendocrine and inflammatory processes [21, 22] which in turn impact both physical and mental health. Individual health risk (e.g. smoking, alcohol consumption) could link perceived racial discrimination and health, as means of coping with or avoiding discrimination [23, 24].

Although a growing number of studies have investigated the link between racial discrimination and health, there are still areas where more research is required. In the 2015 racism meta-analysis of almost 300 studies, only 9% of the data included were prospective [16]. The authors aimed to compare the effect sizes of the cross-sectional and prospective studies included in their review but were unable to conduct this analysis for the physical outcomes data, emphasising the need for more prospective studies on physical health outcomes in particular.

Further, the literature is dominated by United States (US)-based studies drawn from convenience samples [12, 16]. In the latest racism and health meta-analysis, over one third of the articles included were drawn from student samples and only nine (2.7%) of the included studies were UK-based [16]. This is important as the makeup of ethnic minority groups in the UK differs from that of the US, with those of South Asian backgrounds forming the largest minority group [25]. In addition, all of the UK studies were cross-sectional in nature and focused on mental health, with physical outcomes such as the number of physical illnesses [26] and self-rated health [27] included in only two of the studies.

To date, one UK study has assessed the relationship between racial discrimination and health prospectively. In an analysis of the UK Household Longitudinal Study (UKHLS), the authors found that those who reported racial discrimination had poorer mental functioning scores 4 years later [28]. They also reported a dose-response relationship between the experience of racial discrimination and mental health, with those who reported racial discrimination at more than one timepoint over a 3-year period experiencing a greater deterioration in mental functioning.

Overall, there is a dearth of prospective evidence on the link between racial discrimination and health in UK samples, particularly in relation to physical health outcomes.

To address these gaps in the literature, the present study set out to assess cross-sectional and prospective associations between racial discrimination and health in a large community-dwelling UK population cohort. Specifically, we were interested in psychological distress, mental functioning and life satisfaction, as indicators of mental health, as well as self-rated health and physical functioning as markers of physical health, along with limiting longstanding illness as an indicator of impairment. We hypothesised that those who perceived racial discrimination would have poorer health across all measures both cross-sectionally and prospectively.

## Methods

### Study population

The current study uses data from UKHLS [29]. The study began in 2009/10 (wave 1) with follow-ups yearly. This study uses data from waves 1 (2009/10) and 3 (2011/12) of the data collection. The UKHLS consists of a representative sample of the UK population, as well as an ethnic minority boost sample [25, 30]. In this study we use data from ‘extra 5 minutes sample’ of over 8000 individuals who had an additional 5 min of questions on issues of importance to ethnicity research including discrimination. The majority of this sample are drawn from ethnic minority groups (*n* = 6722), in addition to a smaller comparison group of white participants (*n* = 1428) [25]. We restricted our analyses to those who provided information on racial discrimination at wave 1 (*n* = 5707) and self-reported being of non-white ethnicity (*n* = 4883). The participants included in our study were significantly older (*p* = 0.002) and were less likely to have an educational qualification (*p* < 0.001) than those who did not provide data for the study. They were also more likely to be male (*p* < 0.001) and of South Asian ethnicity (*p* < 0.001) The groups did not differ on income (*p* = 0.136). All participants provided fully informed written consent and the University of Essex Ethics Committee granted ethical approval for UKHLS.

### Racial discrimination

To measure perceived discrimination, participants were asked whether in the past 12 months, they had (a) felt unsafe, (b) avoided going to or being in, (c) been insulted, called names, threatened or shouted at, or (d) been physically attacked in 7 different settings 1) At school/college/work, 2) On public transport, 3) At or around bus or train stations, 4) In a taxi, 5) Public buildings such as shopping centres or pubs, 6) Outside on the street, in parks or other public places, or 7) At home. If they answered yes to any one of these questions, a follow-up question asked them to choose an attribution for the discrimination from a list of categories including ethnicity, nationality, age, and sex among others. Participants could choose multiple settings and attributions for the perceived discrimination. Those who attributed any experience of discrimination to their ethnicity or nationality are treated as cases of perceived racial discrimination in our analyses. Those who did not perceive any form of discrimination serve as the comparison group in our analyses. Those who reported other (non-racial) forms of discrimination were not included in the analysis. This measure has been used in previous investigations to look at the link between perceived discrimination and health outcomes [28, 31, 32].

### Mental health outcomes

We included 3 mental health measures at waves 1 (2009/10) and 3 (2011/12). Psychological distress was assessed using the General Health Questionnaire (GHQ)-12 [33], in line with previous studies [31, 32]. This tool has been validated as a screening tool to detect psychological distress in community samples [34]. This measure involved ratings of 12 statements including whether the participant had “*Been able to enjoy your normal day to day activities*” or whether they “*Felt constantly under strain*” with binary response options (yes/no). After totalling, the overall score ranged from 0 (least distressed) to 12 (most distressed). The Cronbach’s alpha for the scale was 0.99.

The 12-item short-form health survey (SF-12) mental component summary score was used to measure limitations caused by emotional, mental health and social functioning issues [35], in keeping with previous studies [31, 32]. This tool has been validated for use as a measure of mental functioning in community samples [35, 36]. Items included ratings of feelings experienced over the past 4 weeks such as “*Have you felt downhearted or blue*?” or *“Accomplished less than you would like”*. A total score ranging from 0 (low functioning) to 100 (high functioning) was derived using standard methods [37]. The Cronbach’s alpha for this scale was 0.98.

One item was used to assess participants’ life satisfaction by asking them how satisfied they were with their “life overall”, on a scale from 1 (completely dissatisfied) to 7 (completely satisfied) [38]. Single item measures of life satisfaction are widely used in survey studies [39] This measure has been used in previous investigations to assess the link between discrimination and life satisfaction [31, 32].

### Impairment outcome

Self-reported limiting longstanding illness at waves 1 (2009/10) and 3 (2011/12) was used as measure of impairment. It was measured using one item *“Do you have any long-standing physical or mental impairment, illness or disability?...mean [ing] anything that has … or is likely to trouble you over a period of at least 12 months”* with response options of yes or no. Self-reported limiting longstanding illness has been investigated in relation to perceived discrimination in other studies [40, 41].

### Physical health outcomes

We included 2 measures of physical health that were assessed at waves 1 (2009/10) and 3 (2011/12). The SF-12 physical component summary score was used to measure limitations caused by deficits in physical functioning [35]. Participants were ask*ed “Does your health now limit you a lot, limit you a little or not limit you at all?”* in activities such *“climbing stairs”* or *“moving a table, pushing a vacuum cleaner, bowling or playing golf”.* Overall scores were derived using standard methods ranging from 0 (low functioning) to 100 (high functioning) [37]. The Cronbach’s alpha for the scale was 0.98. This tool has been validated for use as a measure of physical functioning in community samples [35, 36].

A single item was used to assess self-rated health: “*Would you say your health is … poor/fair/good/very good/excellent?”* In keeping with earlier work [31, 32, 42] self-rated health was dichotomised with 0 being “good/very good/excellent” and 1 being “poor/fair”. This single item measure has been shown to have good predictive validity for health outcomes [42].

### Covariates

Our analyses included covariates that are likely relevant to racial discrimination and physical and mental health. All covariates were assessed at wave 1. Age in years was included as a continuous variable. Self-reported sex was included and coded as male/female. Socioeconomic status is an important contributor to racial disparities in health [43]. Racial discrimination can compound these inequalities. Therefore, we included education as a 3-level variable, coded as 1 “university degree”, 2 “high school qualification” and 3 “no qualification”. Equivalised monthly household income was computed by dividing total household net income by the modified Organization for Economic Cooperation and Development (OECD) equivalence scale to account for the effects of household size and composition [44]. The UKHLS samples the 5 main ethnic minority groups in the UK [25, 30]: Indian, Pakistani, Bangladeshi, Black African and Black Caribbean. Participants were asked *“What is your ethnic group?”* with response options standardised in line with the England and Wales 2011 Census [25]. Response options also accounted for those of *“mixed backgrounds”.* We included ethnicity as a 6-level variable with these 5 main UK minority groups and 1 additional category of non-white individuals from a range of other minority backgrounds including Chinese, Arab and mixed ethnic backgrounds among others. For our sensitivity analysis, we collapsed ethnicity into a 3-level variable with Indian, Pakistani and Bangladeshi participants coded as “South Asian” Black African and Black Caribbean participants coded as “Black” and other non-white participants coded as “Other”.

### Statistical analyses

The characteristics of those who did and those who did not report racial discrimination at wave 1 were compared using Chi-squared tests for categorical variables and independent samples t-tests for continuous variables. Associations between racial discrimination and the mental and physical health measures were assessed using linear regression for continuous outcomes and binary logistic regression for categorical outcomes. For the mental health analyses, psychological distress, mental functioning and life satisfaction were the outcome variables. For the impairment analysis limiting longstanding illness was the outcome variable. For the physical health analyses, physical functioning and self-rated health were the outcome variables. Age, sex, household income, education and ethnicity at wave 1 were adjusted for in all analyses. Baseline (wave 1) score/status on the relevant outcome variable was included as an additional covariate in prospective analyses. Only those with complete case information at wave 1 (*n* = 4883) and wave 3 (*n* = 2833) were included in the analyses. We tested for interactions between racial discrimination and age, sex, income, education or ethnicity on the mental and physical health outcomes at both waves 1 and 3. No significant effects were detected. Thus, interaction terms were not included in our final reported models.

Results from linear regression analyses are presented as unstandardized B and 95% confidence intervals (95% CI). Results from binary logistic regression analyses are presented as odds ratios (ORs) and 95% CI. The level of significance was set at *p* < 0.05. Unstandardized Bs and ORs rather than *p* values should be used to determine the strength of associations. All analyses were conducted using SPSS v.24.

### Sensitivity analyses

To test the robustness of our findings, we conducted three sets of sensitivity analyses. In our first, we investigated whether a certain type of discriminatory experience (i.e. feeling unsafe, avoiding somewhere, being insulted or attacked) contributing to the measure of racial discrimination was driving the results. We tested this by removing each type of discriminatory experience from the exposure variable in turn, as has been done in previous investigations [31, 32, 40]. In the second sensitivity analysis, we assessed whether participants who were lost to follow-up differed from those who provided data at both waves, and tested whether this influenced the findings by conducting the cross-sectional analyses (wave 1) including only those who provided follow-up data at wave 3. In our final sensitivity analysis, we assessed whether the associations between racial discrimination and our health outcomes varied depending on ethnic group (South Asian, Black or Other), as there is currently limited evidence in this area outside of the US context [16].

## Results

A total of 4883 participants were included in our analysis and of these 998 (20.4%) reported ethnicity (*n* = 854) or nationality (*n* = 144) discrimination. The characteristics of the sample at wave 1 in relation to racial discrimination are displayed in Table 1. Those who perceived racial discrimination were younger on average and were more likely to hold a university degree than those who did not perceive racial discrimination. There were no differences in sex or income, but reports of racial discrimination did vary by ethnic group. Those in the Indian (23.3%) and in the Other ethnic group (24%) were most likely to report experiences of racial discrimination. Further detail on the types of racial discrimination and the settings in which the racial discrimination occurred for the different ethnic groups can be found in Supplementary Table 1.

**Table 1:** Associations between racial discrimination and sociodemographic factors at wave 1 (2009/10)

	No racial discrimination (***n*** = 3885)	Racial discrimination (***n*** = 998)	***p***
Age (years)	38.55 (15.60)	36.98 (13.32)	0.001
16–24	793 (20.4%)	174 (17.4%)	
25–34	991 (25.5%)	311 (31.2%)	
35–44	904 (23.3%)	257 (25.8%)	
45–54	593 (15.3%)	151 (15.1%)	
55+	604 (15.5%)	105 (10.5%)	
Sex (% men)	1900 (48.9%)	474 (47.5%)	0.426
Household income (£)	1195.71 (1020.57)	1220.60 (889.97)	0.481
£0–499	572 (14.7%)	138 (13.8%)	
£500–999	1486 (38.2%)	356 (35.7%)	
£1000–1499	909 (23.4%)	221 (22.1%)	
£1500–1999	439 (11.3%)	145 (14.5%)	
£2000+	479 (12.3%)	138 (13.8%)	
Education (% yes)			0.001
University Degree	1372 (35.3%)	474 (47.5%)	–
School qualification	1673 (43.1%)	412 (41.3%)	–
No qualification	840 (21.6%)	112 (11.2%)	–
Ethnicity			0.001
Indian	702 (76.7%)	213 (23.3%)	–
Pakistani	609 (79.6%)	156 (20.4%)	–
Bangladeshi	635 (85.5%)	108 (14.5%)	–
Black Caribbean	464 (82.4%)	99 (17.6%)	–
Black African	580 (80.6%)	140 (19.4%)	–
Other	895 (76.0%)	282 (24.0%)	–

Data are presented as means (SD) and n (%)

### Racial discrimination and mental health

The descriptive characteristics of the sample in relation to health outcomes are displayed in Table 2. The mental health findings from the regression analyses are displayed in the upper panel of Table 3. Cross-sectionally, those who reported racial discrimination had greater psychological distress (*B* = 1.11, 95% CI 0.88; 1.34, *p* < 0.001), poorer mental functioning (*B* = − 3.61; 95% CI -4.29; − 2.93, *p* < 0.001) and lower life satisfaction (*B* = − 0.40, 95% CI -0.52; − 0.27, *p* < 0.001), than those who did not report racial discrimination, independent of covariates.

**Table 2:** Characteristics of the racial discrimination groups by health outcomes

	Wave 1				Wave 3			
	n	No racial discrimination	N	Racial discrimination	n	No racial discrimination	n	Racial discrimination
**Mental health measures**								
Psychological distress								
Mean score (SE)	2486	1.57 (0.06)	715	2.68 (0.10)	1163	1.75 (0.08)	370	2.27 (0.14)
Mental functioning								
Mean score (SE)	3848	50.98 (0.16)	994	47.36 (0.31)	1605	49.17 (0.23)	485	47.43 (0.41)
Life satisfaction								
Mean score (SE)	2475	5.16 (0.03)	712	4.76 (0.06)	1158	4.91 (0.04)	376	4.77 (0.08)
**Impairment measure**								
Limiting longstanding illness								
% (SE)	3884	20.3 (0.01)	996	28.3 (0.01)	2245	22.9 (0.01)	586	26.1 (0.01)
**Physical health measures**								
Physical functioning								
Mean score (SE)	3848	50.71 (0.15)	994	49.85 (0.29)	1605	49.74 (0.21)	485	49.29 (0.37)
Self-rated health								
% (SE)	3884	18.6 (0.01)	998	23.4 (0.01)	2245	21.3 (0.01)	588	24.0 (0.01)

*SE* Standard error

Possible scores on the psychological distress scale range from 0 to 12, possible scores on the mental functioning and physical functioning scales range from 0 to 100, and the life satisfaction scale scores range from 0 to 7

**Table 3:** Cross-sectional and prospective associations between racial discrimination and health outcomes

	Wave 1		Wave 3	
	No racial discrimination	Racial discrimination	No racial discrimination	Racial discrimination
**Mental health outcomes**				
Psychological distress				
Coeff. [95%CI]	Ref	1.11 [0.88; 1.34]***	Ref	0.52 [0.20; 0.85]**
Mental functioning				
Coeff. [95%CI]	Ref	−3.61 [−4.29; −2.93]***	Ref	−1.77 [− 2.70; −0.83]***
Life satisfaction				
Coeff. [95%CI]	Ref	− 0.40 [− 0.52; − 0.27]***	Ref	− 0.15 [− 0.32; 0.03]
**Impairment outcome**				
Limiting longstanding illness				
OR [95%CI]	1.00 (Ref)	1.78 [1.49; 2.13]***	1.00 (Ref)	1.31 [1.01; 1.69]*
**Physical health outcomes**				
Physical functioning				
Coeff. [95%CI]	Ref	−0.86 [−1.50; − 0.27]**	Ref	− 0.45 [− 1.29; 0.39]
Self-rated health				
OR [95%CI]	1.00 (Ref)	1.50 [1.24; 1.82]***	1.00 (Ref)	1.30 [1.00; 1.69]*

All analyses are adjusted for age, sex, household income, education and ethnicity. Prospective analyses are additionally adjusted for baseline status/score

*Coeff* unstandardized B coefficient, *CI* confidence interval; *OR* odds ratio

*p < 0.05, **p < 0.01, ***p < 0.001

In prospective analyses, those who perceived racial discrimination had greater psychological distress 2 years later than those who did not perceive racial discrimination, independent of covariates and baseline psychological distress (*B* = 0.52, 95% CI 0.20; 0.85, *p* = 0.002). We detected an association between racial discrimination and poorer mental functioning (*B* = − 1.77; 95% CI -2.70; − 0.83, *p* < 0.001), independent of covariates and mental functioning at wave 1. In adjusted analyses, those who reported racial discrimination had slightly lower life satisfaction than those who did not report racial discrimination at follow-up (means = 4.77 vs 4.91), but this difference did not reach statistical significance (*p* = 0.102).

### Racial discrimination, impairment and physical health

The impairment and physical health results are displayed in the lower panel of Table 3. The cross-sectional findings suggest that independent of covariates, participants who perceived racial discrimination were significantly more likely on average to report having a limiting longstanding illness (OR = 1.78; 95% CI 1.49; 2.13, *p* < 0.001), and were more likely on average to rate their health as fair/poor (OR = 1.50; 95% CI 1.24; 1.82, *p* < 0.001) than those who did not perceive racial discrimination. Those who reported racial discrimination also had significantly poorer physical functioning (*B* = − 0.86; 95% CI -1.50; − 0.27, *p* = 0.008) than those who did not report racial discrimination in adjusted analyses.

In prospective analyses, those who reported racial discrimination were significantly more likely on average to have a limiting longstanding illness 2 years later than those who did not report racial discrimination, independent of covariates and limiting longstanding illness at baseline (OR = 1.31; 95% CI 1.01; 1.69, *p* = 0.039). A greater proportion of those who reported racial discrimination rated their health as fair/poor on average at follow-up than those who did not report racial discrimination (OR = 1.30; 95% CI 1.00; 1.69, *p* = 0.048) in adjusted analyses. However, we failed to detect a prospective adjusted association between racial discrimination and physical functioning (*p* = 0.290).

### Sensitivity analyses

In the first sensitivity analysis, removing each of the discriminatory experiences from the measure of racial discrimination in turn did not alter any of the cross-sectional results (Table 4, upper panel). Prospectively, the association between racial discrimination and all the mental health measures and limiting longstanding illness remained the same regardless of the type of discriminatory experience removed from the measure (Table 4, lower panel). For self-rated health, the association was fairly robust to the type of discriminatory experience, but was slightly attenuated when “feeling unsafe” was removed from the racial discrimination variable (*p* = 0.133). Again, for the most part, no significant prospective associations were detected for physical functioning except when “feeling unsafe” was removed from the racial discrimination variable (*p* = 0.027).

**Table 4:** Sensitivity analysis- Racial discrimination measure removing one type of discriminatory experience in turn

		Model 1 (excluding feeling unsafe)	Model 2 (excluding avoiding someplace)	Model 3 (excluding being insulted)	Model 4 (excluding being attacked)
**Cross-sectional analyses (wave 1)**					
Psychological distress	Coeff. [95%CI]	1.22 (0.96; 1.48)***	1.10 (0.87; 1.34)***	1.16 (0.90; 1.41)***	1.11 (0.88; 1.34)***
Mental functioning	Coeff. [95%CI]	−3.53 (−4.30; −2.75)***	− 3.64 (− 4.33; − 2.95)***	−3.90 (− 4.65; − 3.16)***	−3.61 (− 4.30; − 2.93)***
Life satisfaction	Coeff. [95%CI]	−0.43 (− 0.57; − 0.30)***	−0.40 (− 0.52; − 0.27)***	−0.39 (− 0.53; − 0.25)***	−0.39 (− 0.52; − 0.27)***
Limiting longstanding illness	OR [95%CI]	1.82 (1.51; 2.24)***	1.72 (1.43; 2.06)***	1.69 (1.39; 2.05)***	1.78 (1.49; 2.13)***
Physical functioning	Coeff. [95%CI]	−0.75 (− 1.47; − 0.03)*	−0.79 (− 1.43; − 0.15)*	−1.20 (− 1.90; − 0.49)***	−0.90 (− 1.54; − 0.26)**
Fair/poor self-rated health	OR [95%CI]	1.59 (1.28; 1.96)***	1.48 (1.22; 1.79)***	1.57 (1.27; 1.94)***	1.51 (1.23; 1.83)***
**Prospective analyses (wave 3)**					
Psychological distress	Coeff. [95%CI]	0.37 (0.01; 0.73)*	0.52 (0.20; 0.85)**	0.62 (0.27; 0.97)***	0.51 (0.19; 0.83)**
Mental functioning	Coeff. [95%CI]	−1.33 (− 2.38; −0.28)*	−1.80 (− 2.74; − 0.86)***	−2.14 (− 3.16; − 1.13)***	−1.71 (− 2.65; − 0.78)***
Life satisfaction	Coeff. [95%CI]	−0.16 (− 0.35; 0.04)	−0.12 (− 0.30; 0.06)	−0.13 (− 0.33; 0.06)	−0.14 (− 0.31; − 0.04)
Limiting longstanding illness	OR [95%CI]	1.34 (1.01; 1.78)*	1.37 (1.06; 1.77)*	1.34 (1.02; 1.78)*	1.33 (1.04; 1.73)*
Physical functioning	Coeff. [95%CI]	− 1.07 (− 2.02; −0.12)*	−0.42 (− 1.27; 0.43)	−0.50 (− 1.43; 0.43)	−0.51 (− 1.35; 0.33)
Fair/poor self-rated health	OR [95%CI]	1.25 (0.93; 1.68)	1.33 (1.02; 1.72)*	1.46 (1.10; 1.93)**	1.32 (1.02; 1.72)*

All analyses are adjusted for age, sex, household income, education and ethnicity. Prospective analyses are additionally adjusted for baseline status/score

Model 1 excludes “*felt unsafe at some place*” from the measure of racial discrimination; Model 2 excludes “*avoided some place*”; Model 3 excludes ““*was insulted at some place”*; and Model 4 excludes “*was attacked at some place*”

*Coeff* unstandardized B coefficient, *CI* confidence interval, *OR* odds ratio, *SE* standard error

*p < 0.05, **p < 0.01, ***p < 0.001

Possible scores on the psychological distress scale range from 0 to 12, possible scores on the mental functioning and physical functioning scales range from 0 to 100, and the life satisfaction scale scores range from 0 to 7

In the second sensitivity analysis (Supplementary Table 2), cross-sectional physical and impairment (lower panel) and mental health (upper panel) findings for those who provided complete data at wave 3 were similar to the full-sample at wave 1.

In our final sensitivity analysis (Supplementary Table 3), we assessed whether the associations between racial discrimination and our health outcomes varied depending on ethnic group (South Asian, Black, Other). For the cross-sectional analyses, the findings for psychological distress and mental functioning did not vary by ethnic group. However, for life satisfaction (*B* = − 0.23; 95% CI -0.47; 0.02, *p* = 0.069), limiting longstanding illness (OR = 1.34; 95% CI 0.93; 1.92, *p* = 0.113), physical functioning (*B* = 0.42; 95% CI -0.84; 1.68, *p* = 0.511), and self-rated health (OR = 1.01; 95% CI 0.67; 1.53, *p* = 0.955) the findings for the Black group were non-significant, with lower point estimates than when the ethnic groups were combined in the main analysis. For the prospective analyses, there was no group difference for the impairment and physical health outcomes. However, the findings for psychological distress (*B* = 0.32; 95% CI -0.18; 0.82, *p* = 0.207), and mental functioning (*B* = − 1.37; 95% CI -2.83; 0.09, *p* = 0.065), were not significant for the South Asian group, with lower point estimates than in the combined model. Interestingly, for life satisfaction, those in the Other ethnic group had significantly lower life satisfaction at wave 3 (*B* = − 0.39; 95% CI -0.69;-0.08, *p* = 0.013), with greater point estimates than in the combined model. This finding remained non-significant for the South Asian and Black groups.

## Discussion

In this large UK-based prospective sample of ethnic minority participants, we detected associations between racial discrimination and poorer health. Cross-sectionally, those who reported racial discrimination had a greater likelihood on average of limiting longstanding illness and poor self-rated health, than those who did not report racial discrimination. Racial discrimination was associated greater psychological distress, lower life satisfaction, and poorer physical and mental functioning. In prospective analyses, those who reported racial discrimination had a greater likelihood on average of limiting longstanding illness and poor self-rated health than those who did not report racial discrimination. Racial discrimination was associated with greater psychological distress and poorer mental functioning over a two-year follow-up period, regardless of baseline health. No significant prospective associations with physical functioning or life satisfaction were detected.

To our knowledge, this is the first prospective UK-based study to investigate both mental and physical health outcomes in relation to racial discrimination. One earlier analysis of the UKHLS found that those who reported racial discrimination had poorer mental functioning over a 1–4 year follow-up period [28]. The current study also found a prospective association between racial discrimination and poor mental functioning. Our study builds upon previous findings by additionally showing that this association is independent of baseline mental functioning. We also observed a prospective association with psychological distress, another marker of mental health, with those reporting racial discrimination experiencing an increase in psychological distress over time. We did not detect a prospective association between racial discrimination and poorer life satisfaction. Mean scores trended in this direction but the association did not reach statistical significance. A 2015 longitudinal analysis of the US-based Health and Retirement Study with over 6000 participants also failed to detect a prospective association between racial discrimination and decreases in life satisfaction [45], and pooled analyses have been unable to investigate prospective associations with life satisfaction due lack of sufficient evidence [12, 16]. A possible explanation for this null finding, consistent with earlier work, is that racial discrimination is more strongly associated with negative mental health outcomes such as psychological distress than with positive outcomes such as life satisfaction [12, 16]. Another potential reason for these findings relates to duration of follow-up, as review evidence suggests that a recent experience of racial discrimination may be more strongly associated with poor mental health and more weakly related to life satisfaction measures [16]. Our follow-up period of 2 years was relatively short which may have contributed to these results.

Reviews in the field [16, 17] have highlighted the need for more prospective evidence, particularly for physical health outcomes [16]. We found that participants who reported racial discrimination were more likely to report having a limiting longstanding illness and poorer self-rated health, independent of baseline status. Meta-analytic evidence has demonstrated an association between racism and poor general health and worse physical health outcomes [16]. We built upon this predominately US-based data (a considerable portion of which used convenience sampling) to demonstrate prospective associations between racial discrimination and physical health outcomes in a representative sample of UK adults from ethnic minority groups. We failed to observe a prospective association between perceived racial discrimination and physical functioning, although participants who reported racial discrimination had slightly lower physical functioning scores prospectively than those who did not report racial discrimination. This lack of association may indicate that ongoing experiences of racial discrimination had already made an impact on physical functioning at the time of wave 1 survey, limiting the scope for further significant decreases in this measure over time, particularly as we took baseline physical functioning into account in our analyses. Another possibility, is that the etiological period involved for a decline in physical functioning may differ from that of mental functioning [14]. These outcomes were measured using the same tool (SF-12) but only mental functioning was significantly associated with racial discrimination over the follow-up period.

Review evidence based on US data suggests that associations between racial discrimination and health may vary depending on ethnic group [16]. In our sensitivity analysis, the cross-sectional results for life satisfaction and impairment and physical health outcomes were non-significant for the Black group. Prospectively the findings for psychological distress and mental functioning were non-significant for the South Asian group. Whereas, life satisfaction was found to significantly decline for the Other group over the follow-up period. Taken together these results suggest associations with health outcomes are strongest for South Asian and Other groups cross-sectionally, while prospectively racial discrimination appears to most consistently impact mental health outcomes in Black and Other ethnic groups. These findings should be interpreted with caution due to the likelihood that some of our analyses were underpowered.

In our cross-sectional analyses, we found that those who perceived racial discrimination had poorer mental health, with greater psychological distress, poorer mental functioning and lower life satisfaction. Previous work in UKHLS has demonstrated a cross-sectional association with psychological distress using pooled data across three waves of data collection [46]. To our knowledge no prior UK-based work has reported on cross-sectional associations with poor mental functioning and low life satisfaction. These findings are consistent with earlier work in other countries [12, 16, 45].

We detected links between racial discrimination and poor physical health and impairment. Specifically, we found that those who reported racial discrimination had poorer self-rated health, poorer physical functioning scores and a greater likelihood of having a limiting longstanding illness than those who did not report racial discrimination. Earlier work using the 1993/1994 UK-based Fourth National Survey of Ethnic Minorities survey reported associations between perceived racial discrimination and poor self-rated health [27, 47] and limiting longstanding illness [47]. Our more recent findings from 2009/2010 suggest that these deleterious associations remain an issue for minorities in the UK.

We detected stronger associations between racial discrimination and health for cross-sectional than for prospective comparisons, in keeping with earlier evidence [16]. However, cross-sectional work cannot determine whether reports of racial discrimination stimulate poor mental and physical health or whether perceptions of racial discrimination are a manifestation of feeling suboptimal mentally or physically. Our prospective findings therefore add to the field in establishing that racial discrimination predicts poor mental and physical outcomes prospectively, net of baseline associations, supporting the hypothesis that racial discrimination has adverse consequences for future health.

With regard to the pathways through which racial discrimination negatively impacts health, there are several possibilities that could help explain our results. One mechanism linking racial discrimination and health may be through the dysregulation of stress-related biological processes. In response to perceived chronic discrimination, stress processes may be frequently activated, which over time may result in disturbances across multiple biological systems, in line with the theory of allostatic load [20]. Review evidence indicates discrimination is associated with heightened cardiovascular responses to stress [11, 21], though it is unclear whether this translates into an increased risk for clinical hypertension [48]. Another biological mechanism that may link discrimination and health is through activation of the hypothalamic-pituitary-adrenal (HPA) axis. Several reviews have linked racial discrimination [21, 49, 50] with changes in various cortisol parameters, which in turn have been linked with poorer mental and physical health [51, 52]. Deleterious changes in other biological processes such as heightened inflammation [22] and alterations in DNA methylation of stress-related genes [53] have been linked with discrimination in recent studies. Alterations in these stress-related biological processes offer a plausible link to negative changes in physical [54, 55] and, mental health outcomes [51, 56]. Racial discrimination has also been associated with disturbances in neurobiological processes, with alterations observed in brain areas such as the anterior cingulate cortex, prefrontal cortex and amygdala which overlap with pathways associated with poor mental health [57].

Individual health risk (e.g. smoking, alcohol consumption etc.) could link perceived racial discrimination and poor mental and physical health, either as a method of coping with the negative psychological effect of perceiving racial discrimination (e.g. excessive alcohol consumption as a coping mechanism) or as a barrier to engaging in healthy behaviours (e.g. avoiding a health service perceived to be discriminatory). Racial discrimination has been associated with smoking [23, 58, 59], excessive alcohol consumption [23, 60], as well as substance abuse [61, 62]. Review evidence has linked discrimination with poor sleep [63] as well as weight gain in prospective studies [24]. This individual health risk offers a plausible indirect pathway linking racial discrimination with both poor mental [64, 65], as well as physical health outcomes [66].

Another possibility at the broader structural level is that racial discrimination may impact health through differential access to societal resources such as education, employment, welfare and criminal justice [14, 15]. In the UK, a 2016 report documented persistent ethnic disparities in educational attainment, employment, access to fair pay and adequate housing, as well the over-representation of ethnic minorities in the criminal justice system [4]. Further, data from this report highlight inequalities in access to healthcare among ethnic minority groups [4]. While meta-analytic evidence indicates that racial discrimination is associated with more negative patient experiences of health services, as well as delaying/not getting healthcare and lack of treatment uptake [19]. As these factors are social determinants of health in of themselves [13–15], they may act as a pathway through which perceptions of racial discrimination can act to negatively influence health.

The results of the current study need to be assessed in terms of strengths and limitations. There is a dearth of prospective evidence on the link between racial discrimination and health in UK samples, particularly in relation to physical health. Our large sample of ethnic minority participants allowed us to examine changes in mental and physical health over 2 years, and demonstrated both cross-sectional and prospective associations. We also adjusted statistically for factors that potentially confound associations, including age, sex, socioeconomic status and ethnicity. Although controlling for covariates does not tease out the complexity of the relationships between perceived racial discrimination and these sociodemographic characteristics [43]. For example, socioeconomic status contributes to racial inequalities in health [43], while racial discrimination can compound these disparities and can be conceptualised as an indicator of structural racism [13]; statistical adjusting for socioeconomic status does not capture these relationships.

The study of racism is a complex and contested area of research [67, 68] and our study was not without limitations. Our measure of perceived discrimination was not specifically tailored for racial discrimination, as participants in the could attribute their experience to other forms of discrimination as well (e.g. sexism, ageism). There is evidence that the exposure instrument can influence associations between racism and physical and mental health outcomes [16]*.* Participants were able to attribute multiple reasons for their report of discrimination, which could have helped to avoid priming and this measure has been used to assess racial discrimination in previous work [28]. However, it is possible that measures such as the Schedule of Racist Events scale [69] and the Perceived Racism Scale [70] with more specific items on racist degradation and experiences of racism in personal and professional contexts could have garnered different results. Further, the self-report individual measure of racial discrimination employed in our study does not capture the structural conditions that shape the varied ways in which racial discrimination operates [14]. We only assessed perceived racial discrimination at baseline in this study and did not investigate whether racial discrimination experiences were persistent or changed over time.

Racial discrimination was assessed by self-reports of experiences in the past year and was therefore subject to recall bias. Our findings reflect the perception of racial discrimination rather than objective encounters with racial discrimination. It is possible that objective encounters with racism and perceiving one’s self as the target of racial discrimination might have different consequences for health. Experimental studies involving exposure to discriminatory scenarios have been used to investigate the health impact of objective exposures to racial discrimination. However, these studies may not represent a gold standard for the study of the relationship between discrimination and health, as meta-analytic evidence indicates that exposure to a single negative event in a laboratory setting does not negatively influence health [12].

## Conclusions

In conclusion, this study adds to the field by demonstrating cross-sectional and prospective relationships between racial discrimination and both mental and physical health outcomes. With the rise in racial discrimination in the UK [6] in the aftermath of the Brexit vote [9] our findings highlight the need to reduce racial discrimination, not only to promote equity, but also to potentially benefit mental and physical health and reduce health inequalities.

Racial discrimination is a complex system that involves assigning ethnic groups differential value, which drives disparities in access to power, resources and opportunities [14, 15]. Due to its multi-faceted nature, occurring at both the structural and individual level multiple interventions will be required to tackle this pervasive determinant of health. Historically, raising awareness of racial discrimination has been necessary to promote activism to bring about legislative and social change to improve the position of ethnic minority groups. In terms of public health, there are calls to integrate research about racial discrimination and health into medical teaching in an attempt to tackle structural racism and to highlight the impact racial discrimination has on health [71, 72]. As well as strategies to reduce the pervasiveness of racial discrimination in institutional contexts, action through social media may have benefits for individual health too. The Black Lives Matter campaign is an example of a recent social media movement which has drawn attention to the issue of racial discrimination. There is some evidence that campaigns may provide a source of empowerment, particularly in a time where ethnic minority youth participation in traditional civic engagement activities are in decline [73]. Evidence suggests the Twitter conversation remained Black-led [73] and that the majority of the 40 million plus tweets were supportive of the movement [73, 74]. However, whether social media campaigns positively [73] or negatively impact minority health [75] remains the subject of debate. Further, it should be acknowledged that interventions to educate and raise awareness do not tackle the structural macro-level forces that shape the position of ethnic minorities in society. Although, more challenging to address, work is required to identify socio-political processes that generate racial discrimination so attempts can be made to mitigate its effects. Research into the pathways underlying the link between racial discrimination and health are required to develop policy and to target interventions in this field.

## Supplementary Information

**Additional file 1:** **Supplementary Table 1.** Racial discrimination types and settings by ethnic group. **Supplementary Table 2.** Associations between racial discrimination and health outcomes (complete cases at wave 3). **Supplementary Table 3.** Cross-sectional and prospective associations between racial discrimination and health outcomes stratified by ethnic group

## Data Availability

The UKHLS datasets analysed during the current study are freely available in the UK Data Service repository https://ukdataservice.ac.uk/

## Abbreviations

CI: Confidence Interval
GHQ-12: General Health Questionnaire-12
HPA: Hypothalamic-pituitary-adrenal
OECD: Organization for Economic Cooperation and Development
OR: Odds Ratio
SF-12: Short-form Health Survey-12
UK: United Kingdom
UKHLS: The United Kingdom Household Longitudinal Study
US: United States
