# Supplementary material for: Racial discrimination and health: a prospective study of ethnic minorities in the United Kingdom
Source: BMC Public Health. 2020 Nov 18;20:1652. doi: 10.1186/s12889-020-09792-1 (PMC7672934; doi:10.1186/s12889-020-09792-1)
Supplement: Supplementary file 1 — Additional file 1: Supplementary Table 1. Racial discrimination types and settings by ethnic group. Supplementary Table 2. Associations between racial discrimination and health outcomes (complete cases at wave 3). Supplementary Table 3. Cross-sectional and prospective associations between racial discrimination and health outcomes stratified by ethnic group [file 12889_2020_9792_MOESM1_ESM.docx]

| **Supplementary table 1:** Racial discrimination types and settings by ethnic group | | | | | | | | | |
| --- | --- | --- | --- | --- | --- | --- | --- | --- | --- |
| **Types of racial discrimination** |  | **Overall** | **Indian** | **Pakistani** | **Bangladeshi** | **Black Caribbean** | **Black African** | **Other** | **p value** |
| Felt unsafe at someplace | | 691 (79.5%) | 157 (83.1%) | 115 (81.6%) | 88 (86.3%) | 51 (69.9%) | 89 (76.7%) | 191 (77.0%) | 0.065 |
| Avoided at someplace | | 289 (33.0%) | 71 (37.0%) | 56 (38.6%) | 36 (36.7%) | 21 (26.3%) | 38 (33.6%) | 67 (26.9%) | 0.078 |
| Felt insulted at someplace | | 518 (55.6%) | 96 (47.5%) | 72 (48.3%) | 53 (54.6%) | 62 (68.9%) | 81 (62.8%) | 154 (58.3%) | **0.002** |
| Attacked at someplace | | 72 (7.6%) | 11 (5.4%) | 8 (5.2%) | 14 (13.6%) | 6 (6.7%) | 16 (11.9%) | 17 (6.4%) | **0.032** |
| **Settings** | |  |  |  |  |  |  |  |  |
| School/work | | 119 (11.9%) | 21 (9.9%) | 8 (5.1%) | 9 (8.4%) | 20 (20.2%) | 26 (18.6%) | 35 (12.4%) | **0.001** |
| Public transport | | 337 (33.8%) | 66 (31.0%) | 48 (30.8%) | 38 (35.5%) | 52 (52.5%) | 49 (35.0%) | 84 (29.9%) | **0.002** |
| Bus or train stations | | 311 (31.2%) | 63 (29.6%) | 45 (28.8%) | 38 (35.2%) | 43 (43.4%) | 43 (30.7%) | 79 (28.1%) | 0.086 |
| Taxis | | 81 (8.1%) | 23 (10.8%) | 13 (8.3%) | 13 (12.1%) | 4 (4.0%) | 15 (10.7%) | 13 (4.6%) | **0.029** |
| Public buildings | | 302 (30.3%) | 67 (31.5%) | 42 (26.9%) | 33 (30.8%) | 33 (33.3%) | 39 (27.9%) | 88 (31.3%) | 0.855 |
| On the street | | 731 (73.3%) | 160 (75.1%) | 111 (71.2%) | 80 (74.8%) | 65 (65.7%) | 101 (72.1%) | 214 (75.9%) | 0.433 |
| At home | | 90 (9%) | 21 (9.9%) | 13 (8.3%) | 11 (10.3%) | 6 (6.1%) | 17 (12.1%) | 22 (7.8%) | 0.596 |

Data are presented as numbers (% yes)

| **Supplementary Table 2:**  Associations between racial discrimination and health outcomes (complete cases at wave 3) | | | | | | | | | | | | |  |
| --- | --- | --- | --- | --- | --- | --- | --- | --- | --- | --- | --- | --- | --- |
|  | |  |  |  |  | **Wave 1** |  |  |  |  | **Wave 3** |  | |
|  | |  |  | **n** | **No racial discrimination** | **n** | **Racial discrimination** |  | **n** | **No racial**  **discrimination** | **n** | **Racial discrimination** | |
| **Mental health measures** | | | | | | | | | | | | | |
| Psychological distress | | | |  |  |  |  |  |  |  |  |  | |
|  | Mean score (SE) | | | 1163 | 1.59 (0.08) | 370 | 2.67 (0.15) |  | 1163 | 1.75 (0.08) | 370 | 2.27 (0.14) | |
|  | Coeff. [95%CI] | | |  | Ref |  | 1.08 [0.75; 1.41]*** |  |  | Ref |  | 0.52 [0.20; 0.85]** | |
| Mental functioning | | | |  |  |  |  |  |  |  |  |  | |
|  | Mean score (SE) | | | 1605 | 51.11 (0.24) | 485 | 47.33 (0.45) |  | 1605 | 49.17 (0.23) | 485 | 47.43 (0.41) | |
|  | Coeff. [95%CI] | | |  | Ref |  | -0.36 [-0.53; -0.19]*** |  |  | Ref |  | -0.15 [-0.32; 0.03] | |
| Life satisfaction | | | |  |  |  |  |  |  |  |  |  | |
|  | Mean score (SE) | | | 1158 | 5.15 (0.04) | 376 | 4.79 (0.08) |  | 1158 | 4.91 (0.04) | 376 | 4.77 (0.08) | |
|  | Coeff. [95%CI] | | |  | Ref |  | -0.36 [-0.53; -0.19]*** |  |  | Ref |  | -0.15 [-0.32; 0.03] | |
| **Impairment measure** | | | | | | | | | | | | | |
| Limiting longstanding illness | | | |  |  |  |  |  |  |  |  |  | |
|  | % (SE) | | | 2245 | 21.6 (0.01) | 586 | 31.5 (0.01) |  | 2245 | 22.9 (0.01) | 586 | 26.1 (0.01) | |
|  | OR [95%CI] | | |  | 1.00 (Ref) |  | 1.91 [1.53; 2.39]*** |  |  | 1.00 (Ref) |  | 1.31 [1.01; 1.69]* | |
| **Physical health measures** | | | |  |  |  |  |  |  |  |  |  | |
| Physical functioning | | | |  |  |  |  |  |  |  |  |  | |
|  | Mean score (SE) | | | 1605 | 51.63 (0.22) | 485 | 49.94 (0.40) |  | 1605 | 49.74 (0.21) | 485 | 49.29 (0.37) | |
|  | Coeff. [95%CI] | | |  | Ref |  | -1.69 [-2.58; -0.80]*** |  |  | Ref |  | -0.45 [-1.29; 0.39] | |
| Self-rated health | | | |  |  |  |  |  |  |  |  |  | |
|  | % (SE) | | | 2245 | 19.3 (0.01) | 588 | 25.4 (0.01) |  | 2245 | 21.3 (0.01) | 588 | 24.0 (0.01) | |
|  | OR [95%CI] | | |  | 1.00 (Ref) |  | 1.61 [1.26; 2.05]*** |  |  | 1.00 (Ref) |  | 1.30 [1.00; 1.69]* | |
| All analyses are adjusted for age, sex, household income, education and ethnicity. Prospective analyses are additionally adjusted for baseline status/score.  Coeff = unstandardized B coefficient (white rows), CI = confidence interval, CVD= Cardiovascular disease; OR = odds ratio (grey rows), SE = standard error.  **p*<0.05, ***p*<0.01, ****p* <0.001  Possible scores on the psychological distress scale range from 0-12, possible scores on the mental functioning and physical functioning scales range from 0-100, and the life satisfaction scale scores range from 0-7. | | | | | | | | | | | | |  |

|  | **Supplementary Table 3**  Cross-sectional and prospective associations between racial discrimination and health outcomes stratified by ethnic group | | | | | | |
| --- | --- | --- | --- | --- | --- | --- | --- |
| **Cross-sectional analyses (wave 1)** | | |  | **Black** | **South Asian** | **Other** |  |
| Psychological distress | | Coeff. [95%CI] | | 1 (0.56; 1.44)*** | 1.23 (0.88; 1.58)*** | 1.03 (0.58; 1.47)*** |  |
| Mental functioning | | Coeff. [95%CI] | | -3.33 (-4.70; -1.96)*** | -4.21 (-5.20; -3.22)*** | -2.70 (-3.97; -1.43)*** |  |
| Life satisfaction | | Coeff. [95%CI] | | -0.23 (-0.47; 0.02) | -0.51 (-0.70; -0.32)*** | -0.39 (-0.61; -0.17)*** |  |
| Limiting longstanding illness | | OR [95%CI] | | 1.34 (0.93; 1.92) | 1.85 (1.43; 2.39)*** | 2.03 (1.42; 2.90)*** |  |
| Physical functioning | | Coeff. [95%CI] | | 0.42 (-0.84; 1.68) | -1.05 (-1.97; -0.12)* | -1.51 (-2.71; -0.32)* |  |
| Fair/poor self-rated health | | OR [95%CI] | | 1.01 (0.67; 1.53) | 1.67 (1.27; 2.22)*** | 1.70 (1.19; 2.45)** |  |
| **Prospective analyses (wave 3)** | | | | **Black** | **South Asian** | **Other** |  |
| Psychological distress | | Coeff. [95%CI] | | 0.61 (0.01; 1.22)* | 0.32 (-0.18; 0.82) | 0.82 (0.21; 1.43)** |  |
| Mental functioning | | Coeff. [95%CI] | | -2.05 (-3.81; -0.30)* | -1.37 (-2.83; 0.09) | -1.89 (-3.51; -0.27)* |  |
| Life satisfaction | | Coeff. [95%CI] | | -0.16 (-0.18; 0.51) | -0.23 (-0.50; 0.05) | -0.39 (-0.69; -0.08)* |  |
| Limiting longstanding illness | | OR [95%CI] | | 0.34 (0.84; 2.37) | 1.19 (0.82; 1.73) | 1.40 (0.87; 2.27) |  |
| Physical functioning | | Coeff. [95%CI] | | -0.64 (-2.19; 0.92) | -0.45 (-1.75; 0.85) | -0.57 (-2.10; 0.97) |  |
| Fair/poor self-rated health | | OR [95%CI] | | 1.28 (0.74; 2.22) | 1.13 (0.78; 1.63) | 1.63 (0.97; 2.74) |  |
| All analyses are adjusted for age, sex, household income, education and ethnicity. Prospective analyses are additionally adjusted for baseline status/score.  Coeff = unstandardized B coefficient (white rows), CI = confidence interval, OR = odds ratio (grey rows), SE = standard error.  **p*<0.05, ***p*<0.01, ****p*<0.001  Possible scores on the psychological distress scale range from 0-12, possible scores on the mental functioning and physical functioning scales range from 0-100, and the life satisfaction scale scores range from 0-7. | | | | | | | |
